# Supplementary material for: Cross-talk between Hippo and Wnt signalling pathways in intestinal crypts: Insights from an agent-based model
Source: Comput Struct Biotechnol J. 2020 Jan 10;18:230–40. doi: 10.1016/j.csbj.2019.12.015 (PMC7790739; doi:10.1016/j.csbj.2019.12.015)
Supplement: Supplementary data 1 [file mmc1.pdf]

Cross-talk between Hippo and Wnt signalling pathways in intestinal crypts: insights from an agent-based model.

Supplementary Information

Single-cell modelling

The single-cell modelling we conducted combines the subcellular Wnt/Hippo signalling model, as described by a set of 9 Ordinary Differential Equations (ODEs, Figure S1) and the cell-cycle model, which is described by 5 additional ODEs (Figure S2).

The subcellular signalling model we developed is adapted from a previously published ODE model (1, 2), which describes the dynamics of β-catenin within the crypt epithelial cells, and the effects of signalling (Wnt) on the cell-cycle. The system variables, which represent protein concentrations (in [nM]), are described in the main text and in Figure 1b, with the signalling-dependent rates indicated by the coloured arrows (yellow and pink for Wnt and Hippo dependence, respectively); the parameters used for this signalling subcellular network model, and their descriptions, are included in Table S1. The derivation of the core β-catenin kinetic model can be found in the original publications (1, 2); parameters related to the adhesive, cytoplasmic and transcriptional components of β-catenin kinetics were kept as in the original model (1, 2).

The key difference with the original model is that we included a Hippo-dependent parameter to phenomenologically represent sequestration of free cytoplasmic β-catenin due to Hippo signalling (3). Specifically, our model describes the rate of Hippo-signalling-dependent complex (β-catenin/YAP-P, variable C_H in Equation (5), Figure S1); the latter can reduce the level of free β-catenin within the cell, and hence its translocation to the nucleus and subsequent transcription of cell-cycle genes (Figure 1).

We described the formation of the β-catenin/YAP-P complex as being governed by Michaelis-Menten kinetics (term 1 in Equation (5), Figure S1); also, we assumed that it can localise to the cell-surface forming adhesive complexes with free cytoplasmic β-catenin (term 2 in Equations (5), Figure S1), and that it is ubiquitinated (term 3 in Equations (5), Figure S1). The β-catenin/YAP-P

complex effectively links the Hippo signalling (whose strength is represented by parameter $\hat{p}_H$ in Equation (5), Figure S1) to the level of free cytoplasmic β-catenin (CC, Equation (4), Figure S1), which in turn regulates the amount of transcriptionally active β-catenin (CT, Equation (4), Figure S1). CT positively controls cell proliferation (Equation (3) in Figure S2, see description of the cell-cycle model below). Of note, $\hat{p}_H = p_H(V)$ takes discrete values (and is named p_H) in the single-cell model simulations, where it was varied from 0 to 28000h-1 in 500h-1 intervals (see section below for the definition of $\hat{p}_H$ and its response to cell volume (V) in the agent-based simulations), and KH was varied between 0nM and 20nM in 1nM intervals (see Main text and Figure 2a, b). Parameters and initial conditions for Equations (1-9) (Figure S1) are reported in Tables S1 and S2, respectively. Unlike the original model we assumed that there is only one form (open) of β-catenin and we did not consider the transcription of Wnt-target genes, which do not feedback into the other model variables.

The cell-cycle ODE model we implemented (Figure S2) was proposed in Swat et al. (4) and used in (2); it describes the interactions of 5 molecular components which control the transition of mammalian cells from the G_1 to the S phases of the cell cycle, and includes the following proteins: phosphorylated and unphospohrylated retinoblastoma proteins (pRB_p and pRB, respectively), the transcription factor E2F1, and active and inactive cyclin D kinase complexes ($CycD_a$ and $CycD_i$ respectively). The level of E2F1 has been shown to positively regulate the G_1 to S phase transition (5). In the equations (1-5) in Figure S2, parameters k and φ are non-negative rate constants, parameters K and J are Michaelis-Menten dissociation constants, and C_T is the level of transcriptional β-catenin, as governed by Equation (9) in Figure S1. Thus, the interplay between crypt signalling (Wnt/Hippo) and the cell-cycle is governed by the level of transcriptional β-catenin (C_T), as $CycD_i$ levels (Equation (3), Figure S2) depend on Wnt signalling. As the level of Wnt signalling is decreased, the production of C_T is reduced; this results in a decrease of both CycD expression, and of the downstream cell-cycle effector E2F1, preventing cell transitioning from the G_1 to the S cell-cycle phase. The parameters and initial conditions for the cell-cycle model remained set as in Swat et al. (4); parameters reflect experimentally known features of each molecular component involved in the cell-cycle, with the fitting procedure described in the original publication.

The combined subcellular representation of the Wnt/Hippo signalling and the cell-cycle is therefore comprised of 14 ODEs (combined Equations in Figure S1 and S2). The ODEs were solved using the ODE45 function in MATLAB R2017b, with a timestep of 0.005h, for a total simulation duration of 40h. The length of the G_1 phase of the cell cycle (Figure 2 a, b) was determined by the time taken for the concentration of E2F1 in the cell to exceed 1, as identified in Swat et al. (4).

Agent-based modelling

Our agent-based model was implemented within the Chaste (v. 3.3) modelling framework (6, 7). The subcellular Wnt/Hippo signalling and the cell-cycle model, and related parameters, remained as in the single-cell model. The only differences are the rate $\hat{p}_H$ and the constant K_H (Equation (5) Figure S1). The rate $\hat{p}_H$ is now a function of volume described as follows:

$$\hat{p}_H = p_H(V) = \begin{cases} 0, & V \geq V_{thr} \\ \frac{p_{HMax}}{\Delta}(1 - \frac{V}{CI_{thr}}), & V_{thr}(1-\Delta) \leq V < V_{thr} \\ p_{HMax}, & V < V_{thr}\,(1-\Delta) \end{cases}$$

where V_{thr} is the threshold volume for the onset of contact inhibition (equal to $CI_{thr}\,V_{eq}$), p_{HMax} is the maximal rate of Hippo dependent β-catenin sequestration (set using the single cell model as shown in Figure 2), and Δ sets the volume reduction required to cause complete cell-cycle cessation. The value for Δ is fitted to allow cell-cycle length elongation before cessation, with $\Delta = 0$ effectively causing contact inhibition to occur instantaneously when the cell volume drops below the threshold V_{thr}. Including $\Delta > 0$ maintains the proliferative region towards the crypt base. The process of cell-cycle cessation due to volume reduction has been previously implemented as a switch (8), stopping cell-cycle progression below a set volume, while our implementation results in smoother transitions. The value of CI_{thr} is varied in our agent-based simulations between 0.6 and 0.9 (see Main Text), and p_{HMax} remains fitted as in the single cell analysis (Table S1). The equilibrium cell volume (V_{eq}), calculated as volume of proliferative cells, was set as 0.84, taken from the volume distribution of a relaxed crypt.

In the agent-based model, ODEs were solved using the Sundials CVODE solving tools, which dynamically varies the timestep and solving method depending on the system.

The crypt domain was modelled as an unrolled cylinder, allowing for the projection of cells from the three-dimensional physiological space to a two-dimensional modelling space, containing approximately 14 cells in diameter and 19 cells in hight, as suggested by mouse experimental data (9). The unrolled cylinder representation has periodic boundaries in the X domain, a solid boundary at $Y = 0$ and a sloughing boundary at the top of the crypt ($Y = Y_{\max}$) which removes those cells whose cell centres pass it. An artefact of the 2D cylindrical representation of the cells is that those cells on the base boundary ($Y = 0$) appear significantly smaller, which causes these cells to be more contact-inhibited. To account for this, cells with volumes below 0.15 (calculated from the distribution of equilibrium crypt volumes) were scaled by a factor of 5, such that the volume distribution at the base could match the volume distribution in the rest of the proliferative region of the crypt. The threshold volume for contact inhibition is a variable parameter in our investigation (see above and Main Text).

In agent-based simulations, we coupled the cell-cycle model to the level of transcription complexes C_T (Figure 1b), as in (2) and in the single-cell model described above. The greater Wnt signal at the base of the crypt causes the cell to produce Cyclin D and thus progress through the cell cycle more rapidly, ultimately creating an increasing age gradient over the crypt, and the appearance of a proliferative base of the crypt, as seen experimentally (9). This cell-cycle model was applied to both the externally-imposed gradient (M_E) and division-based (M_I) Wnt models.

Agent-based simulations (Figures 3-5) were carried out with a timestep of 0.01h; before the *in silico* experiments were started, the crypt was allowed to reach equilibrium by simulating an initial 300 hours to allow cell volumes to relax from the initial honeycomb tessellation and the subcellular model to reach equilibrium. Each experiment, of 1000 (simulated) hours duration, was repeated 150 times to provide sufficient data for statistical analysis.

The box plots in Figure 3c, d show the median velocity of the cells at increasing heights up the crypt, with the box representing the 25th and 75th percentiles respectively. The whiskers extend to the most extreme velocities recorded over the course of the 150 repeated experiments. The error bars in Figure 4 represent the mean and standard deviation across the repeated experiments. Mitotic proportions (Figures 3, 4) are defined as the proportion of cells residing in the M phase of the cell cycle, averaged over time, at a given point in the crypt (mean over a region of size 10% of the total

crypt height). Mitotic proportion is thus a measure of the proliferative capacity of the cells, as it indicates that cells are progressing through the cell cycle.

The shaded regions in Figure 5 denote the 95% confidence interval for a binomial distribution with probability p, according to $p \pm z_{0.975}\sqrt{p(1-p)/n}$, where n is the number of simulations conducted, and $z_{0.975}$ is the 97.5 percentile of a standard normal distribution. In the mutant cell simulations, one mutant cell was introduced at the base of the crypt (in a random location along the first row of cells) at the start of the simulation; the washout probability was defined as the proportion of simulations where there were no mutant cells left within the crypt at the end of the experiment (after 1000 hours).

The code for reproducing all simulations is available at https://figshare.com/s/a27badc5b4e0cce1ac6b.

APC-mediated degradation of β-catenin

$$\frac{d}{dt}[D] = s_D[X] - (\tilde{d}_D + \tilde{d}_{DX})[D], \quad (1)$$

$$\frac{d}{dt}[X] = s_X - s_D[X] - \tilde{d}_X[X] + \tilde{d}_{DX}[D], \quad (2)$$

$$\frac{d}{dt}[C_U] = \frac{p_U[D][C_C]}{[C_C]+K_D} + \frac{p_U[D][C_H]}{[C_H]+K_D} - d_U[C_U], \quad (3)$$

Cytoplasmic β-catenin

$$\frac{d}{dt}[C_C] = s_C + d_{CA}[C_A] + d_{CT}[C_T] - (s_{CA}[A] + s_{CT}[T] + d_C)[C_C] - \frac{p_U[D][C_C]}{[C_C]+K_D} - \frac{\hat{p}_H[C_C]}{[C_C]+K_H}, \quad (4)$$

$$\frac{d}{dt}[C_H] = \frac{\hat{p}_H[C_C]}{[C_C]+K_H} - s_{CA}[C_H][A] - \frac{p_U[D][C_H]}{[C_H]+K_D}, \quad (5)$$

E-cadherin-mediated cell-cell adhesion

$$\frac{d}{dt}[A] = s_A + d_{CA}[C_A] - (s_{CA}[C_C] + d_A + s_{CA}[C_H])\,[A], \quad (6)$$

$$\frac{d}{dt}[C_A] = s_{CA}[C_C][A] - d_{CA}[C_A] + s_{CA}[C_H][A], \quad (7)$$

Transcription of Wnt-target cells

$$\frac{d}{dt}[T] = s_T + d_{CT}[C_T] - (s_{CT}[C_C] + d_T)[T], \quad (8)$$

$$\frac{d}{dt}[C_T] = s_{CT}[C_C][T] - d_{CT}[C_T], \quad (9)$$

$$\frac{d}{dt}[Y] = \frac{s_Y[C_T]}{[C_T]+K_T} - d_Y[Y]. \quad (10)$$

Figure S1 Ordinary differential equations of the kinetic model of β-catenin localisation shown in Figure 1, describing the change of β-catenin complex concentrations and their link to cell-cycle progression. C_A, C_C, C_T and C_U are the levels of adhesive-linked β-catenin at the cell surface, cytosolic β-catenin, transcriptional nuclear β-catenin, and β-catenin marked for degradation, respectively. *A*, *T*, and *D* denote the concentrations of molecular species forming complexes with β-catenin at the cell surface (forming adhesive complexes at the adherens junction), within the nucleus, and within the destruction complex, respectively. *X* and *Y* denote the levels of Axin and transcribed Wnt target proteins, respectively. C_H denotes the concentration of β-catenin/YAP complex formed due to Hippo

signalling in the cell. The symbols carrying tildes ($\tilde{d}_X$, $\tilde{d}_{DX}$ and $\tilde{d}_D$) vary in response to Wnt signalling. It was assumed that these parameters are linear functions of Wnt signal (W), which varies from $W = 0$ to $W = 1$ within the prescribed Wnt region (40%/100%). They are described as follows: $\tilde{d}_X = \tilde{d}_X(W) = d_X + \varepsilon_X W$, $\tilde{d}_{DX} = \tilde{d}_{DX}(W) = d_{DX} + \varepsilon_{DX} W$ and $\tilde{d}_D = \tilde{d}_D(W) = d_D + \varepsilon_D W$. The rate $\hat{p}_H$ is a function of cell volume, as described in the text. Parameters are described in the text, and their values are reported in Table S1.

$$\frac{d}{dt}[pRB] = k_1 \frac{[E2F1]}{K_{m1}+[E2F1]} \frac{J_{11}}{J_{11}+[pRB]} \frac{J_{61}}{J_{61}+[pRB_p]} - k_{16}[pRB][CycD_a] + k_{61}[pRB_p] - \phi_{pRB}[pRB], \tag{1}$$

$$\frac{d}{dt}[E2F1] = k_p + k_2 \frac{a^2+[E2F1]^2}{K_{m2}{}^2+[E2F1]^2} \frac{J_{12}}{J_{12}+[pRB]} \frac{J_{62}}{J_{62}+[pRB_p]} - \phi_{E2F1}[E2F1], \tag{2}$$

$$\frac{d}{dt}[CycD_i] = \sigma k_3 [C_T] + k_{23}[E2F1] \frac{J_{13}}{J_{13}+[pRB]} \frac{J_{63}}{J_{63}+[pRB_p]} + k_{43}[CycD_a] - k_{34}[CycD_i] \frac{[CycD_a]}{K_{m4}+[CycD_a]} - \phi_{CycD_i}[CycD_i], \tag{3}$$

$$\frac{d}{dt}[CycD_a] = k_{34}[CycD_i] \frac{[CycD_a]}{K_{m4}+[CycD_a]} - k_{43}[CycD_a] - \phi_{CycD_a}[CycD_a], \tag{4}$$

$$\frac{d}{dt}[pRB_p] = k_{16}[pRB][CycD_a] - k_{61}[pRB_p] - \phi_{pRB_p}[pRB_p]. \tag{5}$$

Figure S2. Ordinary differential equations governing cell-cycle progression, linked to the kinetic model of β-catenin localisation shown in Figure 1 (ODEs in Equations S1) through the levels of C_T which affect CycD level. The model includes 5 molecular components which control the transition of mammalian cells from the G_1 to the S phases of the cell cycle: phosphorylated and unphospohrylated retinoblastoma proteins (pRB_p and pRB, respectively), the transcription factor E2F1, active and inactive cyclin D kinase complexes ($CycD_a$ and $CycD_i$ respectively). To link the subcellular β-catenin model with the cell-cycle model we apply a mitogenic factor, $\sigma = 1/25$ (Equation 3). Further details and parameter values can be found in the original papers (2, 4).

s_A	20 nM/h*	d_A	2 h^{-1}*	d_X	100 h^{-1}*	ε_{DX}	5 h^{-1}*
s_{CA}	250 $(nMh)^{-1}$*	d_{CA}	350 h^{-1}*	d_Y	1 h^{-1}*	ε_D	5 h^{-1}*
s_C	25 nM/h*	d_U	1 h^{-1}*	K_D	5 nM*	Δ	0.2#
s_{CT}	30 $(nMh)^{-1}$*	d_{CT}	750 h^{-1}*	K_T	50 nM*		
s_D	100 h^{-1}*	d_D	5 h^{-1}*	p_U	100 h^{-1}*		
s_T	10 nM/h*	d_{DX}	5 h^{-1}*	K_H	20 nM^		
s_X	10 nM/h*	d_T	0.4 h^{-1}*	p_{Hmax}	26000 h^{-1}^		
s_Y	10 h^{-1}*	d_U	50 h^{-1}*	ε_X	200 h^{-1}*		

Table S1. Table of model parameter values (ODEs in Figure S1). s_A is the basal rate of adhesive molecule formation; s_{CA} is the formation rate of adhesive β-catenin complexes; s_c is the rate of production of β-catenin within the cytosol; s_{CT} is the formation rate of transcriptional β-catenin; s_D is the rate of formation of the destruction complex; s_T is the basal rate of transcriptional molecule production; s_X is the basal rate of Axin production; s_Y is the maximal rate of transcription of Wnt target genes; d_A is the degradation rate for adhesive molecules; d_{CA} is the disassociation rate of adhesive complexes; d_C is the APC independent degradation rate of β-catenin; d_{CT} is the dissociation rate of transcriptional complexes; d_D and ε_D are the coefficients of the Wnt-dependent degradation of the destruction complex (a linear function of Wnt, defined in the legend of Figure S1); d_{DX} and ε_{DX} are the coefficients of the Wnt-dependent disassociation rate for the destruction complex into Axin (idem); d_X and ε_X are the coefficients of the Wnt-dependent degradation rate of Axin (idem); d_T is the degradation rate for transcriptional molecules; d_{CU} is the degradation rate of ubiquitinated β-catenin; d_Y is the rate of transcription of Wnt target proteins; K_D is a saturation term for the ubiquitination of β-catenin by the destruction complex; K_T is the saturation term for the transcription of Wnt target proteins due to the nuclear accumulation of β-catenin; p_U is the association term form the destruction complex and cytoplasmic β-catenin for ubiquitination; K_H is the saturation term for the formation of a YAP-P/β-catenin complex; p_{HMax} is the maximum association rate for YAP-P and β-catenin; Δ sets the volume reduction required to cause complete cell-cycle cessation.* parameters as in (1, 2), ^ parameters fitted in single-cell analysis, # parameter estimated using agent-based modelling, such that the reduction in volume causes cell cycle length elongation before cessation, maintaining the proliferative niche towards the crypt base.

[A]	10 nM	[C_T]	2.54 nM	[X]	0.067 nM
[C_A]	18.14 nM	[D]	0.67 nM	[C_U]	0.45 nM
[C_C]	2.54 nM	[T]	25 nM	[C_H]	0.0 nM

Table S2. Table of initial conditions for subcellular Wnt model described in Figure S1.

References

1. van Leeuwen IMM, Byrne HM, Jensen OE, King JR. Elucidating the interactions between the adhesive and transcriptional functions of β-catenin in normal and cancerous cells. Journal of Theoretical Biology. 2007;247:77-102.

2. van Leeuwen IMM, Mirams GR, Walter A, Fletcher AG, Murray PJ, Osborne J, et al. An integrative computational model for intestinal tissue renewal. Cell Proliferation. 2009;42:617-36.

3. Imajo M, Miyatake K, Iimura A, Miyamoto A, Nishida E. A molecular mechanism that links Hippo signalling to the inhibition of Wnt/β-catenin signalling. EMBO Journal. 2012;31:1109-22.

4. Swat M, Kel A, Herzel H. Bifurcation analysis of the regulatory modules of the mammalian G_1/S transition. Bioinformatics. 2004;20:1506-11.

5. Kel A, Deineko I, Kel-Margoulis O, Wingender E, Ratner V. Modelling of gene regulatory networks of cell cycle control. Role of E2F feedback loops. German Conference on Bioinformatics GCB. 2000:107-114

6. Pitt-Francis J, Pathmanathan P, Bernabeu MO, Bordas R, Cooper J, Fletcher AG, et al. Chaste: A test-driven approach to software development for biological modelling. Computer Physics Communications. 2009;180:2452-71.

7. Mirams GR, Arthurs CJ, Bernabeu MO, Bordas R, Cooper J, Corrias A, et al. Chaste: An Open Source C++ Library for Computational Physiology and Biology. PLoS Computational Biology. 2013;9(3):e1002970..

8. Dunn S, Osborne J, Appleton P, Näthke I. Combined changes in Wnt signaling response and contact inhibition induce altered proliferation in radiation-treated intestinal crypts. Molecular Biology of the Cell. 2016; 27(11), 1863-1874.

9. Sunter JP, Appleton DR, Dé Rodriguez MS, Wright Na, Watson AJ, De Rodriguez MS. A comparison of cell proliferation at different sites within the large bowel of the mouse. Journal of Anatomy. 1979;129:833-42.
